# *trans*-Bis(μ-benzene­thiol­ato-κ^2^ *S*:*S*)bis[chlorido­(tri­phenyl­phosphane-κ*P*)palladium(II)] chloro­form disolvate

**DOI:** 10.1107/S1600536813019806

**Published:** 2013-07-24

**Authors:** Alcives Avila-Sorrosa, Alicia Reyes-Arellano, Juan Manuel Germán-Acacio, Reyna Reyes-Martínez, David Morales-Morales

**Affiliations:** a Departamento de Química Orgánica, IPN, Escuela Nacional de Ciencias Biológicas, Caprio y Plan de Ayala S/N, Colonia Santo Tomás, 11340 México, DF, Mexico; b Ciencias Básicas e Ingeniería, Recursos de la Tierra, Universidad Autónoma Metropolitana, Avenida Hidalgo Poniente, La Estación Lerma, Lerma de Villada, 52006 Estado de México, CP, Mexico; c Instituto de Química, Universidad Nacional Autónoma de México, Circuito Exterior, Ciudad Universitaria, DF 04510, Mexico

## Abstract

The title compound, [Pd_2_Cl_2_(C_6_H_5_S)_2_(C_18_H_15_P)_2_]·2CHCl_3_, contains a centrosymmetric dinuclear palladium complex with the Pd^II^ cation in a slightly distorted square-planar coordination environment. The Pd^II^ cations are bridged by the S atoms of two benzene­thiol­ate ligands with different Pd—S distances [2.2970 (11) and 2.3676 (11) Å]. The coordination of the metal atom is completed by a chloride anion [2.3383 (11) Å] and a tri­phenyl­phosphane ligand [2.2787 (11) Å]. Weak C—H⋯Cl inter­actions are present between complex mol­ecules and the CHCl_3_ solvent mol­ecule. The latter is disordered over two positions in a 0.792 (8):0.208 (8) ratio. The crystal under investigation was found to be twinned by nonmerohedry, with a fraction of 73.4 (1)% for the major twin component.

## Related literature

For related complexes in catalysis reactions, see: Yin & Liebscher (2007 ▶); Frisch & Beller (2005 ▶); Knochel & Singer (1993 ▶); Surry & Buchwald (2008 ▶). For bond lengths in a related complex, see: Estudiante-Negrete *et al.* (2007 ▶).
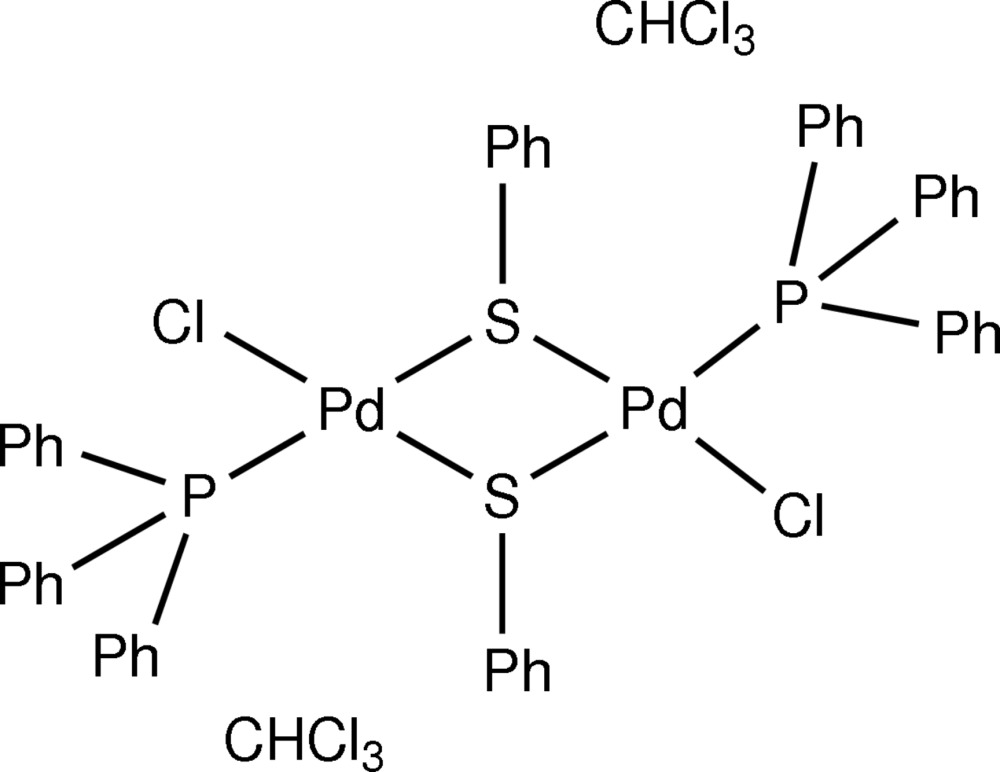


## Experimental

### 

#### Crystal data

[Pd_2_Cl_2_(C_6_H_5_S)_2_(C_18_H_15_P)_2_]·2CHCl_3_
*M* *_r_* = 1265.29
Monoclinic, 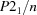

*a* = 10.8343 (11) Å
*b* = 14.2291 (15) Å
*c* = 17.3994 (18) Å
β = 102.095 (2)°
*V* = 2622.8 (5) Å^3^
*Z* = 2
Mo *K*α radiation
μ = 1.27 mm^−1^
*T* = 298 K
0.36 × 0.19 × 0.10 mm

#### Data collection

Bruker SMART APEX CCD area-detector diffractometer
Absorption correction: multi-scan (*TWINABS*; Bruker, 2007 ▶) *T*_min_ = 0.707, *T*_max_ = 0.878
4876 measured reflections
4876 independent reflections
4577 reflections with *I* > 2σ(*I*)

#### Refinement

*R*[*F* ^2^ > 2σ(*F* ^2^)] = 0.039
*wR*(*F* ^2^) = 0.088
*S* = 1.08
4876 reflections
318 parameters
96 restraints
H-atom parameters constrained
Δρ_max_ = 0.55 e Å^−3^
Δρ_min_ = −0.31 e Å^−3^

### 

Data collection: *APEX2* (Bruker, 2007 ▶); cell refinement: *SAINT* (Bruker, 2007 ▶); data reduction: *SAINT*; program(s) used to solve structure: *SHELXL2013* (Sheldrick, 2008 ▶); program(s) used to refine structure: *SHELXL2013*; molecular graphics: *ORTEP-3 for Windows* (Farrugia, 2012 ▶); software used to prepare material for publication: *SHELXL2013* and *PLATON* (Spek, 2009 ▶).

## Supplementary Material

Crystal structure: contains datablock(s) I, global. DOI: 10.1107/S1600536813019806/wm2753sup1.cif

Structure factors: contains datablock(s) I. DOI: 10.1107/S1600536813019806/wm2753Isup2.hkl

Additional supplementary materials:  crystallographic information; 3D view; checkCIF report

## Figures and Tables

**Table 1:** Hydrogen-bond geometry (Å, °)

*D*—H⋯*A*	*D*—H	H⋯*A*	*D*⋯*A*	*D*—H⋯*A*
C25—H25⋯Cl1^i^	0.98	2.79	3.744 (6)	164
C15—H15⋯Cl1^ii^	0.93	2.93	3.650 (5)	135

Symmetry codes: (i) 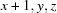; (ii) 
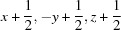
.

**Fig. 1.**
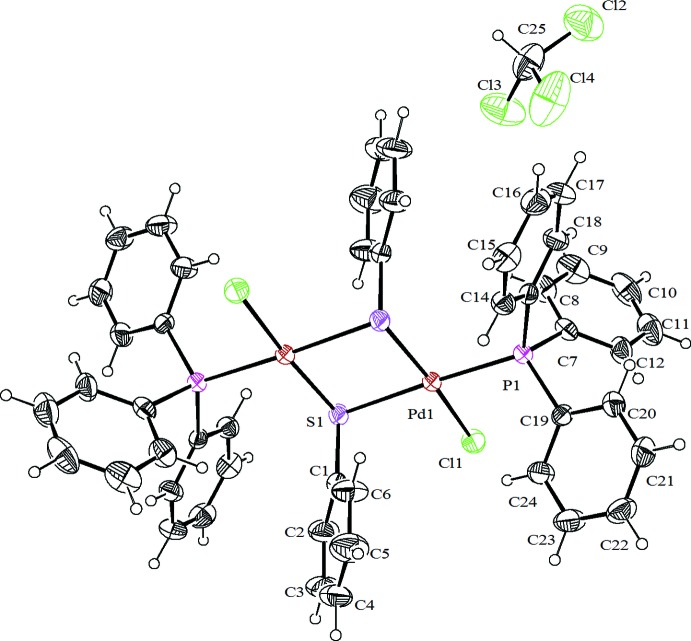
The molecular structure of the title compound, showing the displacement parameters of atoms at the 30% probability level. Only the major component of the disordered solvent molecule is displayed.

**Fig. 2.**
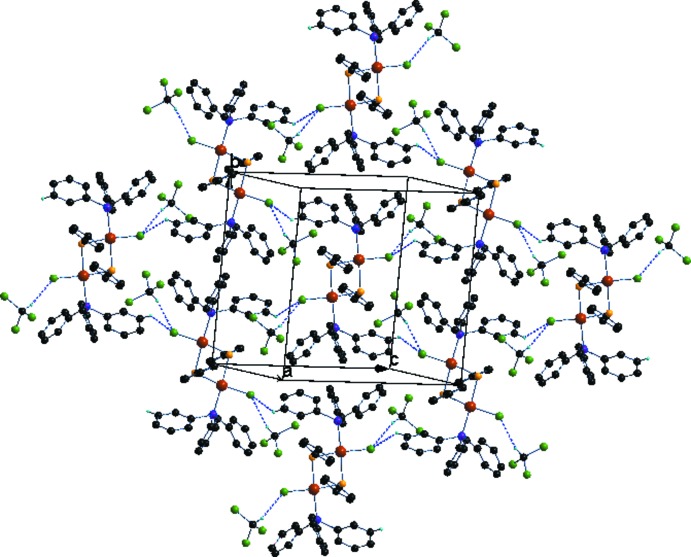
Packing of the molecular entities in the structure of (I). C—H···Cl interactions are shown by dashed lines.

## supplementary crystallographic information

### Comment

Palladium is a transition metal well known for synthetic chemists. Thus, palladium complexes have been extensively studied and have had important applications as catalysts in a plethora of organic transformations, biological applications, material science *etc*. As catalysts, palladium complexes have shown high efficiency in different protocols of cross-coupling reactions for the construction of C—C bonds, *e.g.* through Heck reaction (Yin & Liebscher, 2007), Suzuki–Miyaura coupling (Frisch & Beller, 2005), Negishi reaction (Knochel & Singer, 1993), as well as for the formation of C-heteroatom highlighted *via* Buchwal–Hartwig reaction (Surry & Buchwald, 2008). In this context we report here the structure of the title compound, *trans*-[PdCl(C_6_H_5_S)(C_18_H_15_P)]_2_.2CHCl_3_, (I).

The structure of (I) contains a centrosymmetric dimeric Pd^II^ complex with the Pd^II^ atom within a slightly distorted square-planar coordination environment (Fig. 1). The two Pd^II^ atoms are bridged by two –SC_6_H_5_ ligands, and the coordination environment is completed by one Cl^-^ and one PPh_3_ ligand. The asymmetric unit is composed of half of the complex and one disordered CHCl_3_ solvent molecule; the full molecule is completed by application of inversion symmetry.

The two bridging –SC_6_H_5_ ligands exhibit different Pd1—S1 bond lengths, 2.2970 (11) and 2.3676 (11) Å; these distances are comparable to those found in the structure of the related compound [Pd(PPh_3_)(SC_6_F_5_)(µ-SC_6_F_5_)]_2_ (Estudiante-Negrete *et al.*, 2007). The Pd1—P1 and Pd1—Cl1 bonds lengths are 2.2787 (11) and 2.3383 (11) Å, respectively. The Cl^-^ and the PPh_3_ ligands are arranged in a mutually *trans* conformation.

The complex molecules are packed in rows parallel to [010]. The structure is stabilized by weak C—H···Cl hydrogen-bonding interactions between complex molecules and interstitial solvent molecules (Fig. 2).

### Experimental

To a suspension of PdCl_2_ (0.078 g, 0.442 mmol) and Na_2_CO_3_ (0.050 g, 0.047 mmol) in 15 ml of toluene a solution of 1-ethenyl-[(phenylsulfanyl)methyl]-benzene (isomeric mix 60:40), 1-ethenyl-3-[(phenylsulfanyl)methyl]-benzene and 1-ethenyl-4-[(phenylsulfanyl)methyl]-benzene) (0.100 g, 0.442 mmol) in 5 ml of toluene was added. The resulting mixture was set to reflux for 4 h. After this time the reaction mixture was allowed to cool down to room temperature and filtered. The solid residue was further washed twice with CHCl_3_ (5 ml) attaining a yellow solid. This solid was further treated with PPh_3_ (0.232 g, 0.884 mmol) in 15 ml of CHCl_3_. The reaction mixture was stirred until all solid was dissolved and the resulting solution was filtered through a short plug of Celite. The filtrate was left to evaporate at room temperature yielding yellow crystals of (I) suitable for X-ray diffraction analysis.

### Refinement

H atoms were included in calculated positions (C—H = 0.93 Å for aromatic H) and refined using a riding model with *U*_iso_(H) = 1.2 *U*_eq_ of the carrier atoms. In the refinement five reflections, (1 16 4), (-11 4 5), (-2 15 8), (8 11 5) and (-9 12 5), were considered as disagreeable and were omitted. The CHCl_3_ solvent is disordered over two sets of sites in a 0.792 (8):0.208 (8) ratio. Twinning by non-merohedy was detected in the cell determination and two orientation matrices were determined. The data were then processed and corrected for absorption effects with the *TWINABS* program (Bruker, 2007). The refinement of the *BASF* parameter was 26.6 (1)%, indicating a fraction of 73.4 (1)% for the major twin component.

### Crystal data

**Table:**

[Pd_2_Cl_2_(C_6_H_5_S)_2_(C_18_H_15_P)_2_]·2CHCl_3_	*F*(000) = 1264
*M**_r_* = 1265.29	*D*_x_ = 1.602 Mg m^−^^3^
Monoclinic, *P*2_1_/*n*	Mo *K*α radiation, λ = 0.71073 Å
*a* = 10.8343 (11) Å	Cell parameters from 9960 reflections
*b* = 14.2291 (15) Å	θ = 2.4–25.3°
*c* = 17.3994 (18) Å	µ = 1.27 mm^−^^1^
β = 102.095 (2)°	*T* = 298 K
*V* = 2622.8 (5) Å^3^	Prism, yellow
*Z* = 2	0.36 × 0.19 × 0.10 mm

### Data collection

**Table:**

Bruker SMART APEX CCD area-detector diffractometer	4876 independent reflections
Detector resolution: 0.83 pixels mm^-1^	4577 reflections with *I* > 2σ(*I*)
ω scans	θ_max_ = 25.4°, θ_min_ = 1.9°
Absorption correction: multi-scan (*TWINABS*; Bruker, 2007)	*h* = −13→12
*T*_min_ = 0.707, *T*_max_ = 0.878	*k* = 0→17
4876 measured reflections	*l* = 0→20

### Refinement

**Table:**

Refinement on *F*^2^	96 restraints
Least-squares matrix: full	Hydrogen site location: inferred from neighbouring sites
*R*[*F*^2^ > 2σ(*F*^2^)] = 0.039	H-atom parameters constrained
*wR*(*F*^2^) = 0.088	*w* = 1/[σ^2^(*F*_o_^2^) + (0.0394*P*)^2^ + 2.3327*P*] where *P* = (*F*_o_^2^ + 2*F*_c_^2^)/3
*S* = 1.08	(Δ/σ)_max_ = 0.001
4876 reflections	Δρ_max_ = 0.55 e Å^−^^3^
318 parameters	Δρ_min_ = −0.31 e Å^−^^3^

### Special details

**Table:**

Geometry. All e.s.d.'s (except the e.s.d. in the dihedral angle between two l.s. planes) are estimated using the full covariance matrix. The cell e.s.d.'s are taken into account individually in the estimation of e.s.d.'s in distances, angles and torsion angles; correlations between e.s.d.'s in cell parameters are only used when they are defined by crystal symmetry. An approximate (isotropic) treatment of cell e.s.d.'s is used for estimating e.s.d.'s involving l.s. planes.
Refinement. Refined as a 2-component twin.

### Fractional atomic coordinates and isotropic or equivalent isotropic displacement parameters (Å^2^)

**Table:**

	*x*	*y*	*z*	*U*_iso_*/*U*_eq_	Occ. (<1)
Pd1	0.42887 (3)	0.39507 (2)	0.46354 (2)	0.03549 (10)	
S1	0.38706 (10)	0.55845 (7)	0.45393 (6)	0.0405 (2)	
P1	0.46454 (10)	0.23806 (7)	0.48206 (6)	0.0366 (2)	
Cl1	0.24145 (11)	0.36353 (8)	0.37296 (7)	0.0532 (3)	
C1	0.2753 (4)	0.5666 (3)	0.5147 (3)	0.0471 (11)	
C2	0.1625 (5)	0.6099 (4)	0.4860 (4)	0.0754 (17)	
H2	0.1468	0.6365	0.4361	0.090*	
C3	0.0705 (6)	0.6143 (5)	0.5317 (6)	0.108 (3)	
H3	−0.0064	0.6434	0.5117	0.129*	
C4	0.0927 (7)	0.5769 (5)	0.6043 (6)	0.100 (3)	
H4	0.0312	0.5804	0.6344	0.120*	
C5	0.2049 (8)	0.5339 (5)	0.6339 (5)	0.101 (2)	
H5	0.2205	0.5086	0.6843	0.121*	
C6	0.2949 (5)	0.5280 (5)	0.5889 (4)	0.0769 (17)	
H6	0.3706	0.4974	0.6090	0.092*	
C7	0.4562 (4)	0.1762 (3)	0.3897 (3)	0.0456 (10)	
C8	0.5280 (5)	0.2094 (4)	0.3396 (3)	0.0633 (14)	
H8	0.5778	0.2625	0.3532	0.076*	
C9	0.5273 (7)	0.1650 (5)	0.2693 (3)	0.0813 (18)	
H9	0.5761	0.1882	0.2355	0.098*	
C10	0.4542 (8)	0.0859 (5)	0.2490 (4)	0.087 (2)	
H10	0.4540	0.0553	0.2018	0.104*	
C11	0.3825 (7)	0.0528 (4)	0.2986 (4)	0.0807 (19)	
H11	0.3327	−0.0003	0.2847	0.097*	
C12	0.3829 (5)	0.0970 (3)	0.3692 (3)	0.0595 (13)	
H12	0.3340	0.0737	0.4028	0.071*	
C13	0.6143 (4)	0.2023 (3)	0.5438 (3)	0.0389 (9)	
C14	0.6401 (4)	0.2303 (3)	0.6217 (3)	0.0473 (10)	
H14	0.5818	0.2671	0.6404	0.057*	
C15	0.7505 (5)	0.2047 (4)	0.6720 (3)	0.0587 (13)	
H15	0.7665	0.2239	0.7243	0.070*	
C16	0.8373 (5)	0.1505 (4)	0.6446 (4)	0.0659 (15)	
H16	0.9120	0.1329	0.6784	0.079*	
C17	0.8141 (5)	0.1224 (4)	0.5678 (4)	0.0681 (15)	
H17	0.8727	0.0853	0.5497	0.082*	
C18	0.7030 (4)	0.1492 (3)	0.5166 (3)	0.0519 (12)	
H18	0.6885	0.1313	0.4640	0.062*	
C19	0.3518 (4)	0.1852 (3)	0.5339 (3)	0.0406 (9)	
C20	0.3754 (5)	0.0980 (3)	0.5692 (3)	0.0585 (13)	
H20	0.4467	0.0643	0.5637	0.070*	
C21	0.2941 (6)	0.0605 (4)	0.6125 (3)	0.0665 (14)	
H21	0.3116	0.0023	0.6365	0.080*	
C22	0.1881 (5)	0.1084 (4)	0.6203 (3)	0.0677 (15)	
H22	0.1331	0.0825	0.6490	0.081*	
C23	0.1633 (5)	0.1950 (4)	0.5853 (4)	0.0721 (16)	
H23	0.0913	0.2279	0.5905	0.087*	
C24	0.2451 (5)	0.2335 (3)	0.5425 (3)	0.0564 (12)	
H24	0.2280	0.2923	0.5194	0.068*	
C25	1.0053 (6)	0.1714 (4)	0.3114 (3)	0.0997 (16)	
H25	1.0794	0.2112	0.3300	0.120*	
Cl2	1.0512 (6)	0.0769 (4)	0.2630 (3)	0.1391 (18)	0.792 (8)
Cl3	0.8933 (4)	0.2375 (3)	0.24836 (19)	0.1357 (15)	0.792 (8)
Cl4	0.9460 (7)	0.1371 (4)	0.3917 (2)	0.170 (2)	0.792 (8)
Cl2A	1.0404 (19)	0.0854 (12)	0.2466 (7)	0.105 (4)	0.208 (8)
Cl3A	0.8462 (10)	0.1982 (14)	0.2823 (10)	0.170 (6)	0.208 (8)
Cl4A	1.0302 (13)	0.1208 (9)	0.4047 (4)	0.101 (3)	0.208 (8)

### Atomic displacement parameters (Å^2^)

**Table:**

	*U*^11^	*U*^22^	*U*^33^	*U*^12^	*U*^13^	*U*^23^
Pd1	0.03338 (16)	0.03191 (15)	0.04120 (16)	0.00213 (13)	0.00789 (13)	0.00007 (15)
S1	0.0391 (5)	0.0340 (5)	0.0468 (6)	0.0021 (4)	0.0055 (5)	0.0027 (5)
P1	0.0345 (5)	0.0326 (5)	0.0431 (6)	0.0017 (4)	0.0086 (4)	−0.0023 (5)
Cl1	0.0462 (6)	0.0536 (6)	0.0543 (7)	0.0018 (5)	−0.0021 (5)	−0.0041 (5)
C1	0.038 (2)	0.034 (2)	0.070 (3)	−0.0019 (18)	0.014 (2)	0.000 (2)
C2	0.048 (3)	0.076 (4)	0.105 (5)	0.018 (3)	0.023 (3)	0.022 (4)
C3	0.054 (4)	0.093 (5)	0.186 (9)	0.031 (4)	0.049 (5)	0.033 (6)
C4	0.079 (5)	0.080 (4)	0.164 (8)	0.014 (4)	0.077 (5)	0.011 (5)
C5	0.098 (5)	0.123 (6)	0.096 (5)	0.011 (5)	0.053 (4)	0.014 (5)
C6	0.056 (3)	0.106 (5)	0.077 (4)	0.017 (3)	0.032 (3)	0.019 (4)
C7	0.048 (2)	0.043 (2)	0.045 (2)	0.014 (2)	0.006 (2)	−0.003 (2)
C8	0.076 (4)	0.061 (3)	0.054 (3)	0.007 (3)	0.017 (3)	0.001 (3)
C9	0.097 (5)	0.097 (5)	0.054 (3)	0.025 (4)	0.025 (3)	0.012 (3)
C10	0.118 (6)	0.081 (5)	0.056 (3)	0.033 (4)	0.004 (4)	−0.022 (3)
C11	0.090 (5)	0.061 (4)	0.081 (4)	0.007 (3)	−0.005 (4)	−0.023 (3)
C12	0.065 (3)	0.047 (3)	0.063 (3)	−0.001 (2)	0.004 (3)	−0.018 (2)
C13	0.034 (2)	0.0313 (19)	0.049 (2)	−0.0018 (16)	0.0052 (19)	0.0027 (18)
C14	0.045 (2)	0.046 (2)	0.051 (3)	0.004 (2)	0.009 (2)	0.004 (2)
C15	0.061 (3)	0.066 (3)	0.046 (3)	−0.007 (3)	0.004 (2)	0.009 (2)
C16	0.047 (3)	0.069 (3)	0.076 (4)	0.002 (3)	−0.002 (3)	0.018 (3)
C17	0.045 (3)	0.069 (3)	0.090 (4)	0.018 (3)	0.016 (3)	0.000 (3)
C18	0.044 (3)	0.054 (3)	0.059 (3)	0.009 (2)	0.012 (2)	−0.006 (2)
C19	0.038 (2)	0.041 (2)	0.044 (2)	−0.0053 (17)	0.0111 (19)	−0.003 (2)
C20	0.063 (3)	0.040 (2)	0.077 (3)	0.003 (2)	0.026 (3)	0.005 (3)
C21	0.077 (4)	0.048 (3)	0.077 (4)	−0.006 (3)	0.023 (3)	0.011 (3)
C22	0.058 (3)	0.075 (4)	0.076 (4)	−0.009 (3)	0.029 (3)	0.013 (3)
C23	0.050 (3)	0.083 (4)	0.090 (4)	0.009 (3)	0.030 (3)	0.020 (3)
C24	0.050 (3)	0.058 (3)	0.066 (3)	0.010 (2)	0.022 (2)	0.012 (3)
C25	0.107 (4)	0.104 (4)	0.081 (3)	−0.032 (3)	0.004 (3)	0.014 (3)
Cl2	0.134 (3)	0.134 (3)	0.146 (4)	0.039 (3)	0.022 (3)	0.032 (3)
Cl3	0.129 (3)	0.178 (3)	0.108 (2)	0.060 (2)	0.0416 (18)	0.039 (2)
Cl4	0.248 (6)	0.182 (3)	0.086 (2)	−0.093 (4)	0.049 (3)	0.009 (2)
Cl2A	0.134 (9)	0.126 (8)	0.049 (4)	−0.018 (6)	0.009 (5)	0.012 (4)
Cl3A	0.136 (7)	0.199 (11)	0.155 (11)	0.039 (8)	−0.015 (7)	−0.009 (9)
Cl4A	0.107 (7)	0.130 (7)	0.061 (4)	−0.034 (6)	0.010 (4)	−0.018 (4)

### Geometric parameters (Å, º)

**Table:**

Pd1—P1	2.2787 (11)	C12—H12	0.9300
Pd1—S1^i^	2.2970 (11)	C13—C18	1.383 (6)
Pd1—Cl1	2.3383 (11)	C13—C14	1.384 (6)
Pd1—S1	2.3676 (11)	C14—C15	1.375 (6)
S1—C1	1.770 (5)	C14—H14	0.9300
S1—Pd1^i^	2.2971 (11)	C15—C16	1.376 (8)
P1—C7	1.819 (5)	C15—H15	0.9300
P1—C13	1.821 (4)	C16—C17	1.366 (8)
P1—C19	1.825 (4)	C16—H16	0.9300
C1—C2	1.366 (7)	C17—C18	1.392 (7)
C1—C6	1.378 (8)	C17—H17	0.9300
C2—C3	1.400 (9)	C18—H18	0.9300
C2—H2	0.9300	C19—C24	1.380 (6)
C3—C4	1.346 (11)	C19—C20	1.385 (6)
C3—H3	0.9300	C20—C21	1.380 (7)
C4—C5	1.362 (10)	C20—H20	0.9300
C4—H4	0.9300	C21—C22	1.365 (8)
C5—C6	1.376 (9)	C21—H21	0.9300
C5—H5	0.9300	C22—C23	1.377 (8)
C6—H6	0.9300	C22—H22	0.9300
C7—C8	1.368 (7)	C23—C24	1.385 (7)
C7—C12	1.381 (6)	C23—H23	0.9300
C8—C9	1.376 (8)	C24—H24	0.9300
C8—H8	0.9300	C25—Cl2	1.715 (6)
C9—C10	1.379 (10)	C25—Cl4	1.726 (6)
C9—H9	0.9300	C25—Cl3	1.732 (5)
C10—C11	1.359 (10)	C25—Cl3A	1.733 (7)
C10—H10	0.9300	C25—Cl4A	1.745 (7)
C11—C12	1.379 (8)	C25—Cl2A	1.759 (7)
C11—H11	0.9300	C25—H25	0.9800
			
P1—Pd1—S1^i^	95.42 (4)	C11—C12—C7	119.8 (6)
P1—Pd1—Cl1	90.27 (4)	C11—C12—H12	120.1
S1^i^—Pd1—Cl1	173.83 (4)	C7—C12—H12	120.1
P1—Pd1—S1	175.51 (4)	C18—C13—C14	118.7 (4)
S1^i^—Pd1—S1	83.67 (4)	C18—C13—P1	123.2 (3)
Cl1—Pd1—S1	90.84 (4)	C14—C13—P1	118.1 (3)
C1—S1—Pd1^i^	102.77 (16)	C15—C14—C13	121.2 (4)
C1—S1—Pd1	99.59 (14)	C15—C14—H14	119.4
Pd1^i^—S1—Pd1	96.33 (4)	C13—C14—H14	119.4
C7—P1—C13	105.1 (2)	C14—C15—C16	119.6 (5)
C7—P1—C19	108.8 (2)	C14—C15—H15	120.2
C13—P1—C19	101.47 (19)	C16—C15—H15	120.2
C7—P1—Pd1	111.96 (15)	C17—C16—C15	120.3 (5)
C13—P1—Pd1	117.46 (13)	C17—C16—H16	119.9
C19—P1—Pd1	111.26 (14)	C15—C16—H16	119.9
C2—C1—C6	118.2 (5)	C16—C17—C18	120.2 (5)
C2—C1—S1	118.9 (4)	C16—C17—H17	119.9
C6—C1—S1	122.9 (4)	C18—C17—H17	119.9
C1—C2—C3	120.1 (6)	C13—C18—C17	120.0 (5)
C1—C2—H2	119.9	C13—C18—H18	120.0
C3—C2—H2	119.9	C17—C18—H18	120.0
C4—C3—C2	120.5 (6)	C24—C19—C20	118.6 (4)
C4—C3—H3	119.8	C24—C19—P1	120.7 (3)
C2—C3—H3	119.8	C20—C19—P1	120.5 (4)
C3—C4—C5	120.1 (7)	C21—C20—C19	120.6 (5)
C3—C4—H4	119.9	C21—C20—H20	119.7
C5—C4—H4	119.9	C19—C20—H20	119.7
C4—C5—C6	119.7 (7)	C22—C21—C20	120.5 (5)
C4—C5—H5	120.1	C22—C21—H21	119.7
C6—C5—H5	120.1	C20—C21—H21	119.7
C5—C6—C1	121.4 (6)	C21—C22—C23	119.6 (5)
C5—C6—H6	119.3	C21—C22—H22	120.2
C1—C6—H6	119.3	C23—C22—H22	120.2
C8—C7—C12	119.3 (5)	C22—C23—C24	120.2 (5)
C8—C7—P1	117.8 (4)	C22—C23—H23	119.9
C12—C7—P1	122.9 (4)	C24—C23—H23	119.9
C7—C8—C9	120.6 (6)	C19—C24—C23	120.5 (5)
C7—C8—H8	119.7	C19—C24—H24	119.8
C9—C8—H8	119.7	C23—C24—H24	119.8
C8—C9—C10	119.9 (6)	Cl2—C25—Cl4	111.8 (4)
C8—C9—H9	120.1	Cl2—C25—Cl3	110.6 (4)
C10—C9—H9	120.1	Cl4—C25—Cl3	109.6 (4)
C11—C10—C9	119.6 (6)	Cl3A—C25—Cl4A	108.2 (6)
C11—C10—H10	120.2	Cl3A—C25—Cl2A	107.5 (6)
C9—C10—H10	120.2	Cl4A—C25—Cl2A	107.2 (5)
C10—C11—C12	120.7 (6)	Cl2—C25—H25	108.2
C10—C11—H11	119.6	Cl4—C25—H25	108.2
C12—C11—H11	119.6	Cl3—C25—H25	108.2
			
Pd1^i^—S1—C1—C2	133.2 (4)	C19—P1—C13—C18	120.9 (4)
Pd1—S1—C1—C2	−128.0 (4)	Pd1—P1—C13—C18	−117.6 (4)
Pd1^i^—S1—C1—C6	−49.3 (5)	C7—P1—C13—C14	−172.8 (3)
Pd1—S1—C1—C6	49.5 (5)	C19—P1—C13—C14	−59.4 (4)
C6—C1—C2—C3	0.0 (9)	Pd1—P1—C13—C14	62.1 (4)
S1—C1—C2—C3	177.7 (5)	C18—C13—C14—C15	−1.3 (7)
C1—C2—C3—C4	0.6 (12)	P1—C13—C14—C15	179.0 (4)
C2—C3—C4—C5	−0.3 (13)	C13—C14—C15—C16	0.2 (7)
C3—C4—C5—C6	−0.7 (13)	C14—C15—C16—C17	0.1 (8)
C4—C5—C6—C1	1.4 (12)	C15—C16—C17—C18	0.6 (9)
C2—C1—C6—C5	−1.0 (10)	C14—C13—C18—C17	2.0 (7)
S1—C1—C6—C5	−178.6 (6)	P1—C13—C18—C17	−178.3 (4)
C13—P1—C7—C8	−76.2 (4)	C16—C17—C18—C13	−1.7 (8)
C19—P1—C7—C8	175.8 (4)	C7—P1—C19—C24	−110.5 (4)
Pd1—P1—C7—C8	52.4 (4)	C13—P1—C19—C24	139.1 (4)
C13—P1—C7—C12	102.6 (4)	Pd1—P1—C19—C24	13.4 (4)
C19—P1—C7—C12	−5.5 (4)	C7—P1—C19—C20	73.2 (4)
Pd1—P1—C7—C12	−128.9 (4)	C13—P1—C19—C20	−37.2 (4)
C12—C7—C8—C9	0.3 (8)	Pd1—P1—C19—C20	−162.9 (4)
P1—C7—C8—C9	179.1 (4)	C24—C19—C20—C21	−0.4 (8)
C7—C8—C9—C10	−0.4 (9)	P1—C19—C20—C21	176.0 (4)
C8—C9—C10—C11	0.5 (10)	C19—C20—C21—C22	0.9 (9)
C9—C10—C11—C12	−0.5 (10)	C20—C21—C22—C23	−0.7 (9)
C10—C11—C12—C7	0.4 (9)	C21—C22—C23—C24	0.0 (9)
C8—C7—C12—C11	−0.3 (7)	C20—C19—C24—C23	−0.3 (8)
P1—C7—C12—C11	−179.0 (4)	P1—C19—C24—C23	−176.7 (4)
C7—P1—C13—C18	7.5 (4)	C22—C23—C24—C19	0.5 (9)

Symmetry code: (i) −*x*+1, −*y*+1, −*z*+1.

### Hydrogen-bond geometry (Å, º)

**Table:**

*D*—H···*A*	*D*—H	H···*A*	*D*···*A*	*D*—H···*A*
C25—H25···Cl1^ii^	0.98	2.79	3.744 (6)	164
C15—H15···Cl1^iii^	0.93	2.93	3.650 (5)	135

Symmetry codes: (ii) *x*+1, *y*, *z*; (iii) *x*+1/2, −*y*+1/2, *z*+1/2.
